# Elevated levels of prostaglandin E2 in Yoshida hepatoma and the inhibition of tumour growth by non-steroidal anti-inflammatory drugs.

**DOI:** 10.1038/bjc.1980.56

**Published:** 1980-03

**Authors:** A. Trevisani, E. Ferretti, A. Capuzzo, V. Tomasi

## Abstract

Prostaglandin (PG) E2 biosynthesis in Yoshida hepatoma (AH 130) was evaluated by radioimmunoassay. When hepatoma cells were incubated in vitro, the levels of PGE2 in the medium were similar to those found in hepatocytes for the first 2 h; this was followed by a rapid increase in PGE2 formation, and the 6h incubation levels were 4-fold higher than in hepatocytes. Addition of sodium arachidonate markedly and dose-dependently stimulated PGE2 synthesis; the increase was largely prevented by the addition of indomethacin (1 microM) or L 8027, a prostaglandin synthetase inhibitor. Experiments in vivo indicated that indomethacin treatment of tumour-bearing rats significantly reduced the tumour mass. When rats were injected with PGE2 after receiving the drug, the number of tumour cells was very similar to that of untreated animals. This, as well as the inhibition of tumour growth by acetylsalicylic acid, strongly suggests that the inhibition of PG biosynthesis by anti-inflammatory drugs and the inhibition of tumour proliferation may be closely associated events. It was also found that injections of indomethacin very significantly prolonged survival of hepatoma-bearing rats. Since PGE2 does not appear to affect the cyclic AMP levels of hepatoma cells, it is possible that hepatoma may use PGE2 to subvert the immune system. This could help to explain the effectiveness of anti-inflammatory drugs in the control of tumour growth.


*Br. J. Cancer* (1980) **41**, 341

### ELEVATED LEVELS OF PROSTAGLANDIN $E_2$ IN YOSHIDA HEPATOMA AND THE INHIBITION OF TUMOUR GROWTH BY NON-STEROIDAL ANTI-INFLAMMATORY DRUGS

A. TREVISANI, E. FERRETTI, A. CAPUZZO AND V. TOMASI*

*From the Institute of General Physiology, University of Ferrara and the *Laboratory of General Physiology, University of Bologna, Italy*

Received 26 July 1979 Accepted 29 October 1979

**Summary.—Prostaglandin (PG) $E_2$ biosynthesis in Yoshida hepatoma (AH 130) was evaluated by radioimmunoassay. When hepatoma cells were incubated *in vitro*, the levels of $PGE_2$ in the medium were similar to those found in hepatocytes for the first 2 h; this was followed by a rapid increase in $PGE_2$ formation, and the 6h incubation levels were 4-fold higher than in hepatocytes.**

**Addition of sodium arachidonate markedly and dose-dependently stimulated $PGE_2$ synthesis; the increase was largely prevented by the addition of indomethacin (1 $\mu$M) or L 8027, a prostaglandin synthetase inhibitor.**

**Experiments *in vivo* indicated that indomethacin treatment of tumour-bearing rats significantly reduced the tumour mass. When rats were injected with $PGE_2$ after receiving the drug, the number of tumour cells was very similar to that of untreated animals. This, as well as the inhibition of tumour growth by acetylsalicylic acid, strongly suggests that the inhibition of PG biosynthesis by anti-inflammatory drugs and the inhibition of tumour proliferation may be closely associated events. It was also found that injections of indomethacin very significantly prolonged survival of hepatoma-bearing rats.**

**Since $PGE_2$ does not appear to affect the cyclic AMP levels of hepatoma cells, it is possible that hepatoma may use $PGE_2$ to subvert the immune system. This could help to explain the effectiveness of anti-inflammatory drugs in the control of tumour growth.**

BY SHOWING RAISED LEVELS of prostaglandins (PGs) in tumours and plasma of tumour-bearing patients, Karim (1976) opened up the way to studies which have extended these findings to several human and animal tumours. In addition, using cultured cells, it has been found that transformation by carcinogens (Hong *et al.*, 1977; Levine & Hong, 1977) or by viruses (Hammarstrom, 1977) is followed by increased production of PGs.

It is difficult to correlate these findings with claims that PGs inhibit tumour growth *in vitro* or *in vivo* (Santoro *et al.*, 1976). On the other hand, Plescia *et al.* (1975) have suggested that PGs may be used by tumours to subvert the immune system. This hypothesis also accords with the marked sensitivity of lymphocytes as well as macrophages to PGE which, by increasing cyclic AMP levels, antagonizes mitogen-induced cell proliferation or cytotoxicity (Pelus & Strausser, 1977; Schultz *et al.*, 1978).

The results reported here indicate that the rapidly growing cells of Yoshida hepatoma produce considerable amounts of $PGE_2$ (4-fold more than rat hepatocytes) and that indomethacin or acetylsalicylic acid strongly reduce the growth of the tumour *in vivo* and very significantly prolong the survival of tumour-bearing

Address for correspondence: V. Tomasi, Laboratory of General Physiology, University of Bologna, Via Belmeloro 8, 40126 Bologna, Italy.

rats. Thus, increased $PGE_2$ production seems positively correlated with tumour growth.

### MATERIALS AND METHODS

*Materials.*—$^3$H-prostaglandin $E_2$ (sp. act. 160 Ci/mmol), G-$^3$H-adenosine 3′, 5′-monophosphate (27 Ci/mmol) were obtained from The Radiochemical Centre, Amersham. Anti-prostaglandin $E_2$ serum and bovine serum albumin were products of Sigma Chemical Co., St Louis, Mo, U.S.A. Silicic acid (200–400 mesh) was a product of Merck, Darmstadt, Germany. The prostaglandins were kindly provided by Dr John Pike, The Upjohn Company, Kalamazoo, Mich., U.S.A. Indomethacin (Sigma Chem. Co., U.S.A.) was dissolved in Krebs–Henseleit solution and a few drops of 1N NaOH; L 8027 was provided by S. A. Labaz, Belgium, this being dissolved in ethanol and diluted with the incubation medium. All other chemicals were of the highest reagent grade commercially available.

*Methods.*—Male Wistar rats weighing 80–130 g were used. Yoshida ascites hepatoma AH 130 (about $20 \times 10^6$ cells in 0·5 ml of ascites fluid at 7 days) was injected i.p. The tumour-bearing rats were usually killed 7 days later, and in some experiments 4–11 days later. Cells from ascites fluid, washed twice with 0·9% NaCl, were suspended in Krebs–Henseleit solution, pH 7·4, containing 2% albumin (0·8–1·0 mg protein or 2·5–$3{\cdot}2 \times 10^6$ cells per ml). Such preparations contained >90% viable cells, as judged by the trypan-blue exclusion test and <10% contaminating cells. At the end of the incubations viability was >80%.

Cell suspensions (1 ml) were incubated at 37°C for the indicated times under gassing with 95% $O_2$–5% $CO_2$ in the absence or presence of cold arachidonate (0·1–10·0 μg/ml). After centrifugation supernates were acidified to pH 3·5 with 2N formic acid and PGs were extracted twice with 5 vols ethyl acetate (recovery >95%). The extracts were taken to dryness and PGE was purified by column chromatography using $CHCl_3$/$CH_3OH$ mixtures as described by Salmon & Karim (1975) with a recovery 75%. Radioimmunoassay was carried out as previously described (Bartolini *et al.*, 1978). The incubation system contained in a final volume of 375 μl: 50–100 μl samples or 3–30 pg $PGE_2$; 50 μl antiserum binding 55% of labelled ligand; 25 μl labelled $PGE_2$ (12·5 nCi) and Tris–HCl (0·05M, pH 7·5). Blanks contained no antiserum. Tubes were incubated first for 1 h at 37°C, then for at least 4 h at 4°C. The tubes were placed in an ice bath and 100 μl of freshly prepared albumin-coated charcoal suspension (100 mg/ml 3% bovine serum albumin in Tris–HCl 0·05M, pH 7·5) was quickly added and the tubes were vortexed for 30 s. After standing in ice for 5 min, they were centrifuged at 2000 *g* for 5 min. 0·2ml supernates were counted with 4 ml of Bray solution. Cross-reactivities were as reported elsewhere (Bartolini *et al.*, 1978). In addition, 6-keto-$PGF_{1\alpha}$ cross-reacted 3·5% and $TxB_2$ 0·01%. The method has a sensitivity of 3 pg, an accuracy (recovery of $PGE_2$ added to buffer) >90% and intra- or inter-assay coefficients of variations <10%.

$PGI_2$ was assayed by the platelet aggregation test, as previously described (Tomasi *et al.*, 1978). Aliquots (50 μl) of washed hepatoma cells (10–20 mg protein per ml of buffer) were pre-incubated for 5 min with 0·5 ml human platelet-rich plasma (PRP) and aggregation was induced with 1·5mM Na arachidonate. In other experiments cells were incubated with 0·1mM Na arachidonate for 10 min at 37°C, cells were removed by centrifugation and aliquots of the supernatant solutions were immediately added to 0·5 ml of PRP. Aggregation was induced 2 min later as described above. Cyclic AMP was measured by the method of Brown *et al.* (1971). Proteins were determined by the Lowry method, using bovine serum albumin as standard.

Cell counts were performed on a Model ZBI Coulter Counter (Coulter Electronics, Hialeah, FL.) equipped with a 100μM aperture and a 0·5ml manometer. Yoshida hepatoma cells were counted at settings of 1/amplification = 16 and 1/aperture current = 1/2 with a window of 15–100. The data are usually the mean of 3 observations. The data in Fig. 3 were obtained from a control group (no indomethacin) of 34 rats and an indomethacin-treated group of 36 rats.

Statistical analysis was performed by Student's *t* test.

### RESULTS

### *Prostaglandin synthesis in the hepatoma*

Tumour-cell suspensions (0·8 mg protein or $2{\cdot}5 \times 10^6$ cells per ml) were incubated

FIG. 1.—Time-course of PGE (○——○) and of AMP (×——×) formation in Yoshida hepatoma cells. 1 ml of cell suspension containing 0·8 mg protein in Krebs–Henseleit solution was incubated at 37°C for 2·5–360 min in a shaking bath. The supernatants were then acidified to pH 3·5 with formic acid and PGs were extracted twice with 5 vol of ethylacetate. Extracts were dried and chromatographed as described in the text. PGE was determined by radioimmunoassay. In parallel tubes the cAMP content was evaluated by the protein-binding assay of Brown *et al.* (1971).

at 37°C for 2·5–360 min and the release of $PGE_2$ into the medium was evaluated by radioimmunoassay.

In Fig. 1 it is shown that a slow increase in $PGE_2$ production was followed after 120 min by a rapid increase in the rate of synthesis; even after 360 min incubation the curve failed to reach a plateau. This is in marked contrast to the results using hepatocytes (Bartolini *et al.*, 1978) in which $PGE_2$ production levels off at 60 min and the levels remain even after a 4h incubation (not shown) at least 4-fold lower than those in tumour cells.

In addition, during incubation cyclic AMP levels were determined (Fig. 1). It is clear that while $PGE_2$ synthesis was increasing, no modification of cyclic AMP levels was observed. This, as well as data indicating that cyclic AMP levels are not modified when cells are incubated with $PGE_2$ (1·0–10·0 μg/ml; not shown) strongly suggests that $PGE_2$ does not act directly on the tumour cell, at least as far as cyclic AMP levels are concerned. This does not exclude possible actions on the tumour not mediated by cyclic AMP.

It is well known that endogenous arachidonate is used as a precursor of $PGE_2$ and that addition of this fatty acid stimulates PG synthesis. We found that addition of sodium arachidonate to tumour cells increased $PGE_2$ formation in a dose-dependent fashion. In the presence of 10 μg of this fatty acid, cells were found to produce 14× more $PGE_2$ than in its absence (not shown). A 4-fold increase in $PGE_2$ synthesis was noted in the same conditions using parenchymal cells (Bartolini *et al.*, 1978).

By using a very sensitive biological assay for $PGI_2$ and $TxA_2$, the platelet aggregation test (Moncada *et al.*, 1976), we found that addition of tumour-cell suspensions to human PRP failed both to inhibit or to induce (which is indirect evidence that, at least after short periods of incubation, $TxA_2$ is not formed) arachidonate-induced aggregation (not shown). This, as well as the failure of supernates of cells incubated with Na arachidonate to affect PRP aggregation, strongly suggests that $PGI_2$ is not formed in significant amounts, at least after short-term incubation. We have previously reported (Tomasi *et al.*, 1978) that liver sinusoidal cells were capable, in similar experimental conditions, of inhibiting arachidonate-induced aggregation.

*The effect of non-steroidal anti-inflammatory drugs on the growth rate*

In Fig. 2 it is shown that indomethacin (1·0 μM) *in vitro* markedly inhibits $PGE_2$ formation, especially at the highest dose of arachidonate used. L 8027, a PG synthetase inhibitor, behaved more or less like indomethacin (Fig. 2). Although L 8027 was reported to be a selective $TxA_2$ synthetase inhibitor in platelets (Gryglewski *et al.*, 1977) our data indicate that it may behave also as a potent PG synthetase inhibitor, at least in this hepatoma. It is unlikely that we are measuring $TxB_2$ formation since: (a) the platelet aggregation test gave no indica-

FIG. 2.—The effect of $10^{-6}$M indomethacin (▲) and L 8027 (■) on the biosynthesis of PGE in Yoshida hepatoma cells. Incubations were carried out for 45 min at 37°C. 20 μl of Na arachidonate dissolved in 2% $Na_2CO_3$ was added to each tube. Controls (●) received 20 μl of solvent. Details are described in the text and in the legend to Fig. 1.

FIG. 3.—The effect of indomethacin (●) on the proliferation of Yoshida hepatoma cells. Tumour cells ($20 \times 10^6$) were injected i.p. into 2 groups of rats of the same age (±5 days) and sex. One group received indomethacin twice a day i.p. (1 mg/kg body wt). The second group (○) served as control and received the same amount of the diluent. On the days indicated, 4–6 rats from each group were killed. The ascites fluid (ascites plasma plus tumour cells) was withdrawn by a syringe and the peritoneal cavity opened and thoroughly washed with known volumes of ice-cold 0·9% NaCl. Cells were counted with a Coulter Counter. Tumour mass is expressed as mean cell number ± s.e.

tion of its formation; (b) $TxB_2$ cross-reacts very little (<0·01%) with our antibodies.

These data prompted us to test its effect on tumour growth rate *in vivo*. Two groups of rats were injected with tumour cells and tumour cells plus indomethacin (1 mg/kg body wt) respectively. The drug was then administered twice a day and rats were killed 4–11 days after tumour implants. In Fig. 3 it is shown that in rats injected with indomethacin there was a dramatic reduction in the total number of tumour cells as evaluated by a Coulter Counter, both during the exponential and stationary phases of growth.

To establish whether growth rate is slowed down or proliferation blocked after indomethacin treatment requires a different experimental approach. That this effect of indomethacin is very probably connected with its ability to inhibit PG biosynthesis is supported by experiments showing that acetylsalicylic acid has an effect very like that of indomethacin. This drug, injected i.p. at a dose of 10·0 mg/kg body wt once a day, appears markedly to inhibit tumour mass, at least at the 7th day after tumour implantation (not shown). This low dose is probably effective because it is acting directly on the hepatoma cells in the peritoneal cavity.

Further and more direct evidence was obtained by injecting i.p. $PGE_2$ (1 mg/kg) into tumour-bearing, indomethacin-treated rats. It was found (Fig. 4) that $PGE_2$ was capable of counteracting the inhibition of growth due to the drug, making the growth rate indistinguishable from the controls.

In the Table it is shown that indomethacin treatment very significantly prolongs survival of tumour-bearing animals. The

FIG. 4.—Reversal of inhibition of growth rate in indomethacin-treated rats by $PGE_2$. Indomethacin was administered as in Fig. 3. $PGE_2$ (1 mg/kg body wt) dissolved in saline was injected i.p. once a day. Control group (20 rats) indomethacin-treated (5 rats) and indomethacin plus $PGE_2$-treated (5 rats) were used 6 days after tumour implant.

Bars represent s.e.; $^*P < 0{\cdot}05$ with respect to control; $^{**}P < 0{\cdot}05$ with respect to indomethacin-treated.

TABLE.—*Effect of indomethacin on survival time of tumour-bearing rats*

| Treatment | No. of animals | Survival time ±s.e. (days) |
|---|---|---|
| None | 13 | 8·31 ± 0·99 |
| Indomethacin* | 10† | 12·60 ± 0·90‡ |

* 1 mg/kg body wt twice a day.
† In 3/13 rats the drug completely prevented tumour growth.
‡ $P < 0{\cdot}05$.

data do not take into account observations that indomethacin may sometimes completely overcome the growth of the hepatoma, thus indefinitely prolonging the life-span. Thus 5 rats injected with hepatoma cells and receiving indomethacin were tumour-free after 7 days of treatment, and no toxic effect of the drug was evident after 16 days of treatment.

### DISCUSSION

The data reported indicate that Yoshida hepatoma can be included in the long list of tumours which produce high levels of PGs (Karim, 1976). At present it is difficult to explain why during the first 90 min of incubation tumour cells produce amounts of $PGE_2$ very similar to those produced by isolated hepatocytes, but thereafter there is a marked increase in PG formation.

As far as the significance of this high production of PGs in tumours is concerned, two hypotheses have been more or less explicitly proposed. Thomas *et al.* (1974), after establishing the existence of an inverse relationship between rates of cell proliferation and PGE biosynthesis, suggested that PGE may be directly involved in the inhibition of tumour-cell growth (Santoro *et al.*, 1976). Tumour-growth inhibition by a synthetic analogue of $PGE_2$ has been observed (Santoro & Jaffe, 1979), but Lupulescu (1978) found an enhancement of carcinogenesis after $PGE_2$ administration.

Our data clearly indicate that, at least in Yoshida hepatoma, $PGE_2$ does not appear to act on tumour cells, since cyclic AMP levels do not change during incubation and no change can be demonstrated by incubating cells in the presence of $PGE_2$ (1–10 $\mu$g/ml). On the other hand we have previously found that under similar conditions adrenaline raises cyclic AMP levels at least 2-fold (Tomasi *et al.*, 1974).

We consider more likely the hypothesis proposed by Plescia *et al.* (1975) that tumours may use PGs to subvert the immune system. Such a role of PGE as an intercellular messenger also accords with recent data obtained on liver-cell populations, showing that $PGE_2$ produced mainly in parenchymal cells acts on sinusoidal cells by raising cyclic AMP levels (Bartolini *et al.*, 1978; Tomasi *et al.*, 1978, 1979; Tomasi, 1976).

It is likely that the main targets of locally released $PGE_2$ are cells, such as lymphocytes and (or) macrophages, which are known to participate in the body's defence against tumour cells, and which are known to contain PGE-sensitive adenylate cyclases. After cyclic AMP increase these cells become much less sensitive to various stimuli (Pelus & Strausser, 1977; Schultz *et al.*, 1978).

*The effect of non-steroidal anti-inflammatory drugs on tumour growth*

In most studies showing raised PG levels in tumours, indomethacin or aspirin have been tested as anti-tumour drugs, and in several cases they proved extremely effective. Thus, indomethacin has been shown both to decrease plasma or urinary levels of PGs and to restore normal blood calcium levels both in patients with solid tumours and hypercalcemia (Seyberth *et al.*, 1975; Robertson *et al.*, 1976) and in hypercalcaemic mice bearing a prostaglandin-producing fibrosarcoma (Tashjian *et al.*, 1973). Immunosuppressive mouse tumours treated *in vivo* or *in vitro* with non-steroidal anti-inflammatory drugs lost their capacity to suppress antibody production (Plescia *et al.*, 1975; Grinwich & Plescia, 1977).

Our data clearly show that indomethacin or acetylsalicylic acid at low doses markedly inhibit cell proliferation *in vivo*, an effect which is paralleled by the *in vitro* inhibition of $PGE_2$ biosynthesis. However, we are aware that this correlation has to be considered cautiously, since so many effects of anti-inflammatory drugs apparently unrelated to prostaglandin synthesis have been reported (Flower, 1974; Kantor & Hampton, 1978).

In this respect, the experiments reported in Fig. 4, indicating that injections of $PGE_2$ overcome indomethacin inhibition, strongly suggest that inhibition of PG synthesis and decrease of growth rate are two closely associated events. Unfortunately the most direct approach to this problem (*i.e.* the use of $PGE_2$ antagonists) is hampered by the lack of potency and specificity of the available compounds.

Since $PGE_2$ is well known to have a short life in the circulation, its action may be exerted on lymphocytes or macrophages of the peritoneal cavity. Alternatively, a circulating metabolite of $PGE_2$ with some biological activity may be involved in these effects.

Our data showing that indomethacin very significantly prolongs survival of tumour-bearing rats, as well as similar data recently reported (Lynch & Salomon, 1979; Bennett *et al.*, 1979) are forcing into consideration the use of PG synthetase inhibitors in the combined therapeutic approach to the treatment of some tumours, especially since indomethacin has been reported to be non-immunosuppressive like other types of anti-inflammatory drug (Plescia *et al.*, 1975).

This work was supported in part by a grant from the Consiglio Nazionale delle Ricerche (Progetto finalizzato, Controllo della Crescita Neoplastica). We thank Dr J Pike (Upjohn) for generous gifts of prostaglandins.

### REFERENCES

Bartolini, G., Meringolo, C., Orlandi, M. & Tomasi, V. (1978) Biosynthesis of prostaglandins in parenchymal and nonparenchymal rat liver cells. *Biochim. Biophys. Acta*, **530**, 325.

Bennett, A., Houghton, J., Leaper, D. J. & Stamford, I. F. (1979) Cancer growth, response to treatment and survival time in mice: beneficial effect of the prostaglandin synthesis inhibitor flurbiprofen. *Prostaglandins*, **17**, 179.

Brown, B. L., Albano, J. D. M., Ekins, R. P., Sgherzi, A. M. & Tampion, W. (1971) A simple and sensitive saturation assay method for the measurement of adenosine 3′,5′-cyclic monophosphate. *Biochem. J.*, **121**, 561.

Flower, R. J. (1974) Drugs which inhibit prostaglandin biosynthesis. *Pharmacol. Rev.*, **26**, 33.

Grinwich, K. D. & Plescia, O. J. (1977) Tumor-mediated immunosuppression: Prevention by inhibitors of prostaglandin synthesis. *Prostaglandins*, **14**, 1175.

Gryglewski, R. J., Zmuda, A., Korbut, R., Krecioch, E. & Bieron, K. (1977) Selective inhibition of thromboxane $A_2$ biosynthesis in blood platelets. *Nature*, **267**, 627.

Hammarstrom, S. (1977) Prostaglandin production by normal and transformed 3T3 fibroblasts in cell culture. *Eur. J. Biochem.*, **74**, 7.

Hong, S. L., Wheless, C. M. & Levine, L. (1977) Elevated prostaglandin synthetase activity in methylcholanthrene-transformed mouse BALB/3T3. *Prostaglandins*, **13**, 271.

Kantor, H. S. & Hampton, M. (1978) Indomethacin in submicromolar concentrations inhibits cyclic AMP-dependent protein kinase. *Nature*, **276**, 841.

Karim, S. M. M. (1976) Prostaglandins and tumors. In *Adv. Prostaglandin Res.*, Vol. 2. Ed. S. M. M. Karim. Lancaster: MTP Press. p. 303.

Levine, L. & Hong, S. L. (1977) Analogues of anthracene, phenanthrene, and benzoflavone inhibit prostaglandin biosynthesis by cells in culture. *Prostaglandins*, **14**, 1.

Lupulescu, A. (1978) Enhancement of carcinogenesis by prostaglandins in male Albino Swiss mice. *J. Natl Cancer Inst.*, **69**, 97.

Lynch, N. R. & Salomon, S. C. (1979) Tumor growth inhibition and potentiation of immunotherapy by indomethacin in mice. *J. Natl Cancer Inst.*, **62**, 117.

Moncada, S., Gryglewski, R., Bunting, S. &

VANE, J. R. (1976) An enzyme isolated from arteries transforms prostaglandin endoperoxides to an unstable substance that inhibits platelet aggregation. *Nature*, **263**, 663.

PELUS, L. M. & STRAUSSER, H. R. (1977) Prostaglandins and the immune response. *Life Sci.*, **20**, 903.

PLESCIA, O. J., SMITH, A. H. & GRINWICH, K. D. (1975) Subversion of immune system by tumor cells and role of prostaglandins. *Proc. Natl Acad. Sci. U.S.A.*, **72**, 1848.

ROBERTSON, R. P., BAYLINK, D. J., METZ, S. A. & CUMMINGS, K. B. (1976) Plasma prostaglandin E in patients with cancer with and without hypercalcemia. *J. Clin. Endocrinol. Metab.*, **43**, 1330.

SALMON, J. A. & KARIM, S. M. M. (1975) Methods for analysis of prostaglandins. In *Adv. Prostaglandin Res.*, Vol. 1. Ed. S. M. M. Karim. Lancaster: MTP Press. p. 25.

SANTORO, M. G., PHILPOTT, G. W. & JAFFE, B. M. (1976) Inhibition of tumor growth *in vivo* and *in vitro* by prostaglandin E. *Nature*, **263**, 777.

SANTORO, M. G. & JAFFE, B. M. (1979) Inhibition of Friend erythroleukaemia-cell tumours *in vivo* by a synthetic analogue of prostaglandin $E_2$. *Br. J. Cancer*, **39**, 408.

SCHULTZ, R. M., PAVLIDIS, N. A., STYLOS, W. A. & CHIRIGOS, M. A. (1978) Regulation of macrophage tumoricidal function: A role for prostaglandins of the E series. *Science*, **202**, 320.

SEYBERTH, H. W., SEGRE, G. V., MORGAN, J. L., SWEETMAN, B. J., POTTS, J. T. & OATES, J. A. (1975) Prostaglandins as mediators of hypercalcemia associated with certain types of cancer. *New Engl. J. Med.*, **293**, 1278.

TASHJIAN, A. H., VOELKER, E. F., GOLDHABER, P. & LEVINE, L. (1973) Successful treatment of hypercalcaemia by indomethacin in mice bearing a prostaglandin producing fibrosarcoma. *Prostaglandins*, **3**, 515.

THOMAS, D. R., PHILPOTT, G. W. & JAFFE, B. M. (1974) The relationship between concentration of prostaglandin E and rates of cell replication. *Exp. Cell Res.*, **84**, 40.

TOMASI, V., TREVISANI, A., BIONDI, C. & FERRETTI, E. (1974) Adenosine 3′,5′-cyclic phosphate phosphodiesterase activity of Yoshida hepatoma. *Biochem. Exp. Biol.*, **11**, 175.

TOMASI, V. (1976) Prostaglandin $E_1$ as an intercellular regulator of cyclic AMP levels. *Exp. Cell Biol.*, **44**, 260.

TOMASI, V., BARTOLINI, G., ORLANDI, M., MERINGOLO, C. & BARNABEI, O. (1979) Mechanism of action and biological significance of prostaglandin $E_2$ and prostacyclin in the liver. In *Lipoprotein Metabolism and Endocrine Regulation*. Eds. Hessel & Krans. Amsterdam: Elsevier/North-Holland. p. 279.

TOMASI, V., MERINGOLO, C., BARTOLINI, G. & ORLANDI, M. (1978) Biosynthesis of prostacyclin in rat liver endothelial cells and its control by prostaglandin $E_2$. *Nature*, **273**, 670.


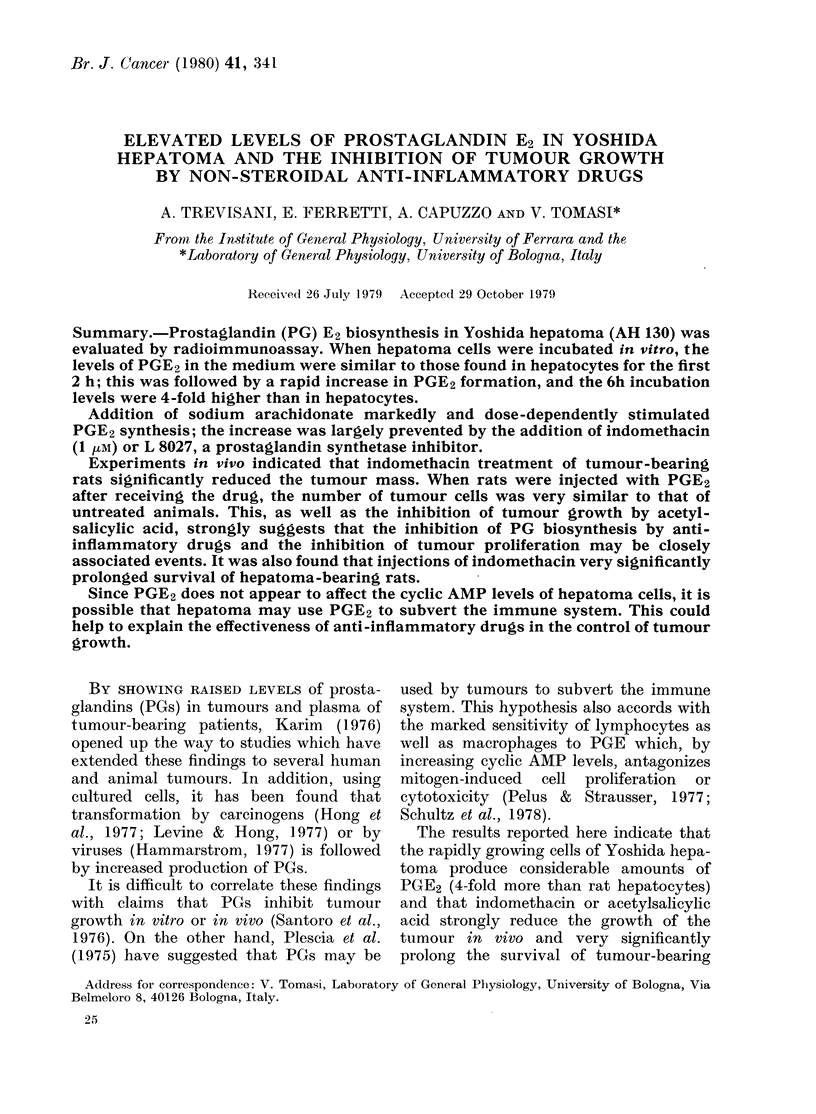

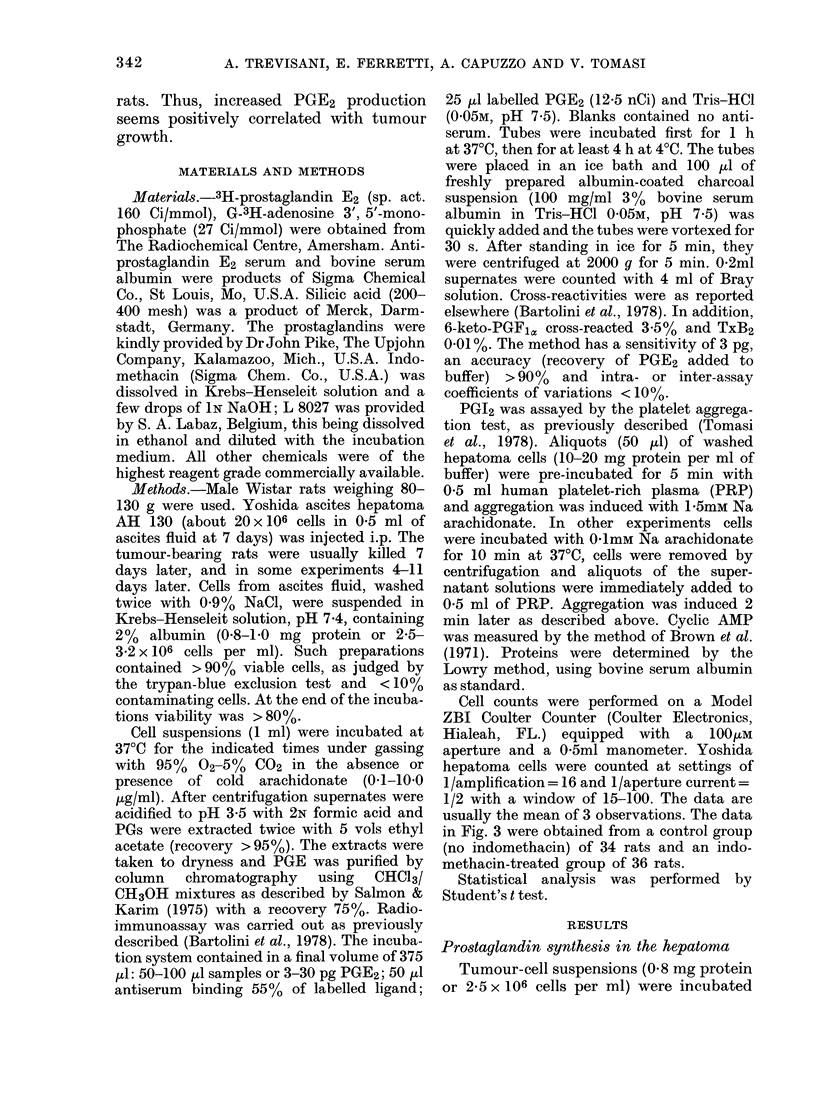

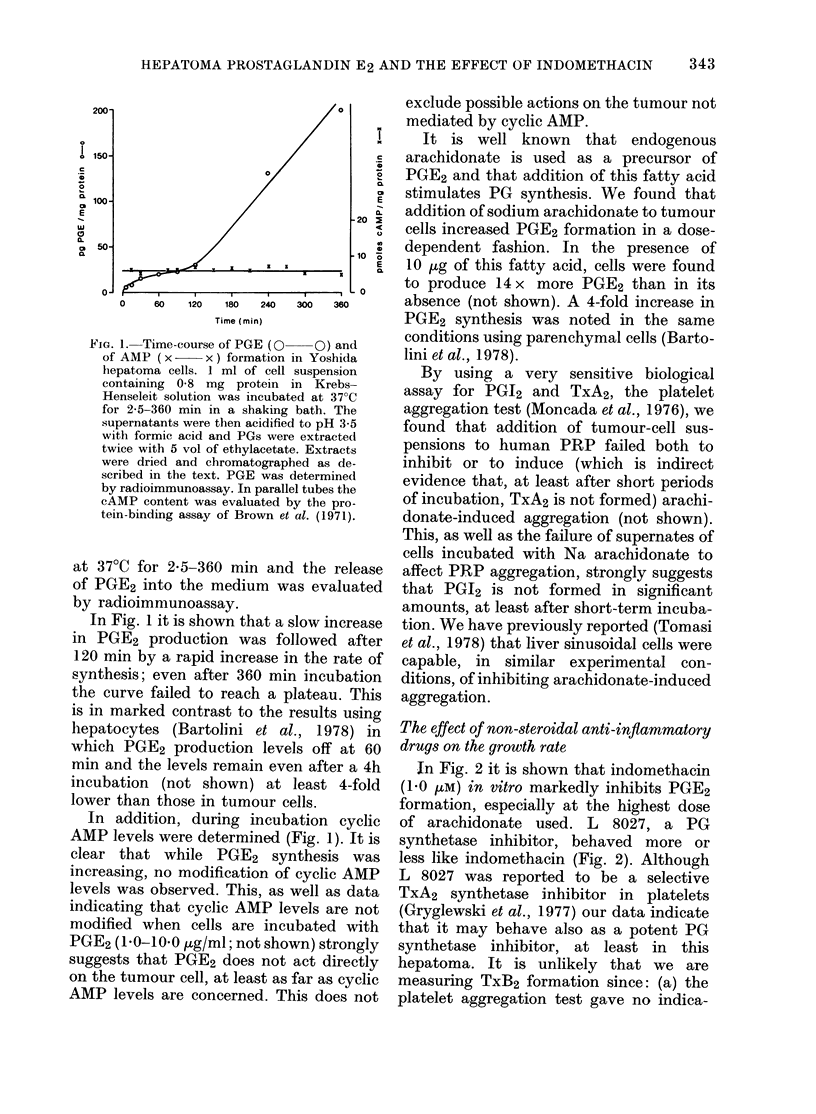

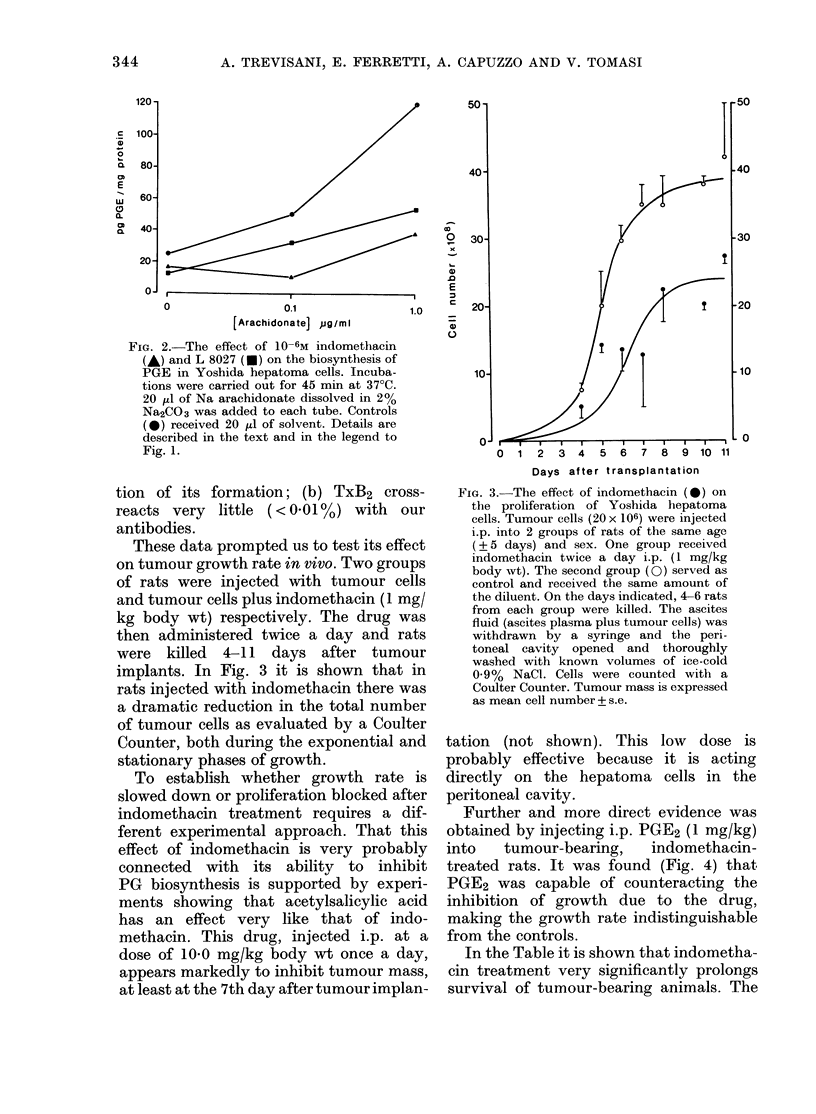

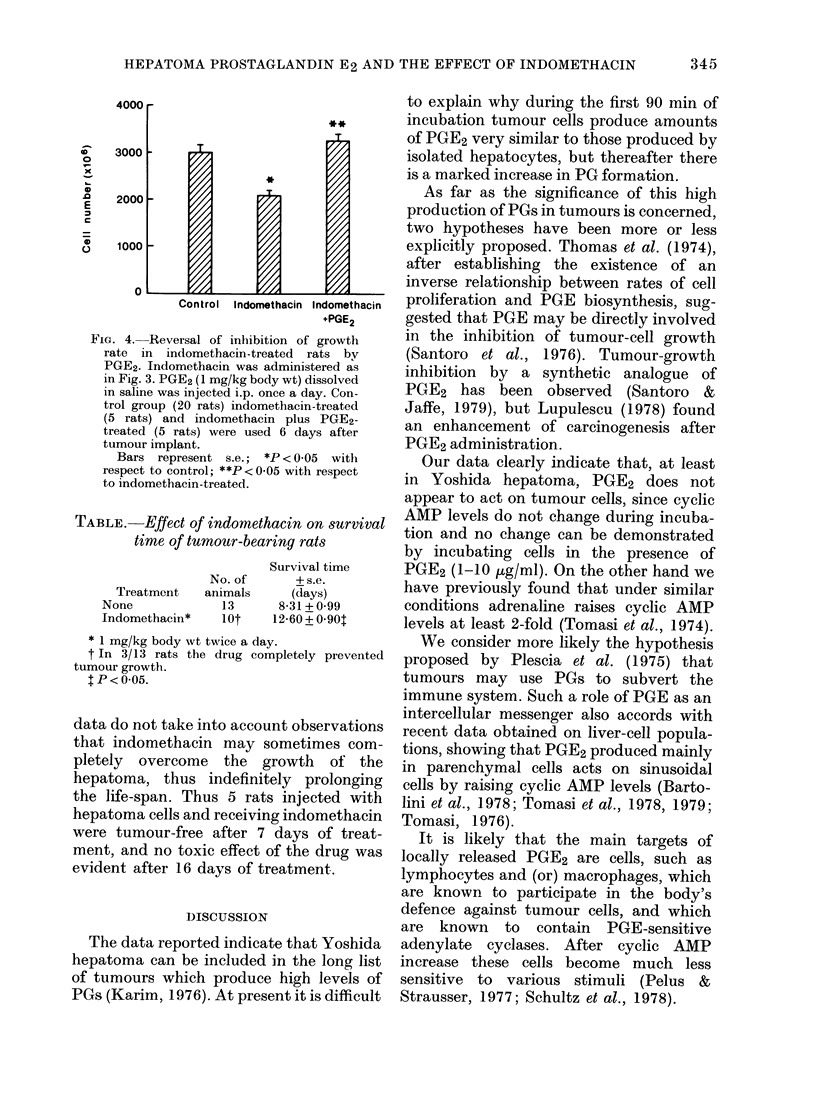

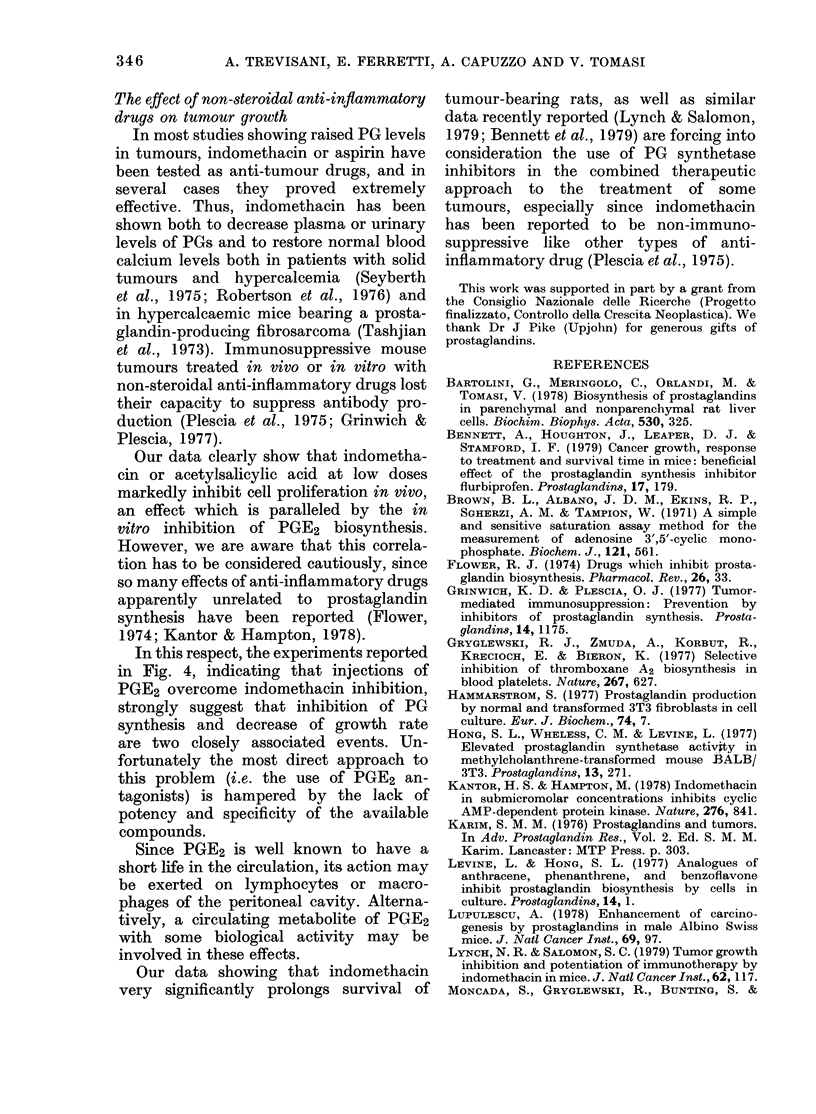

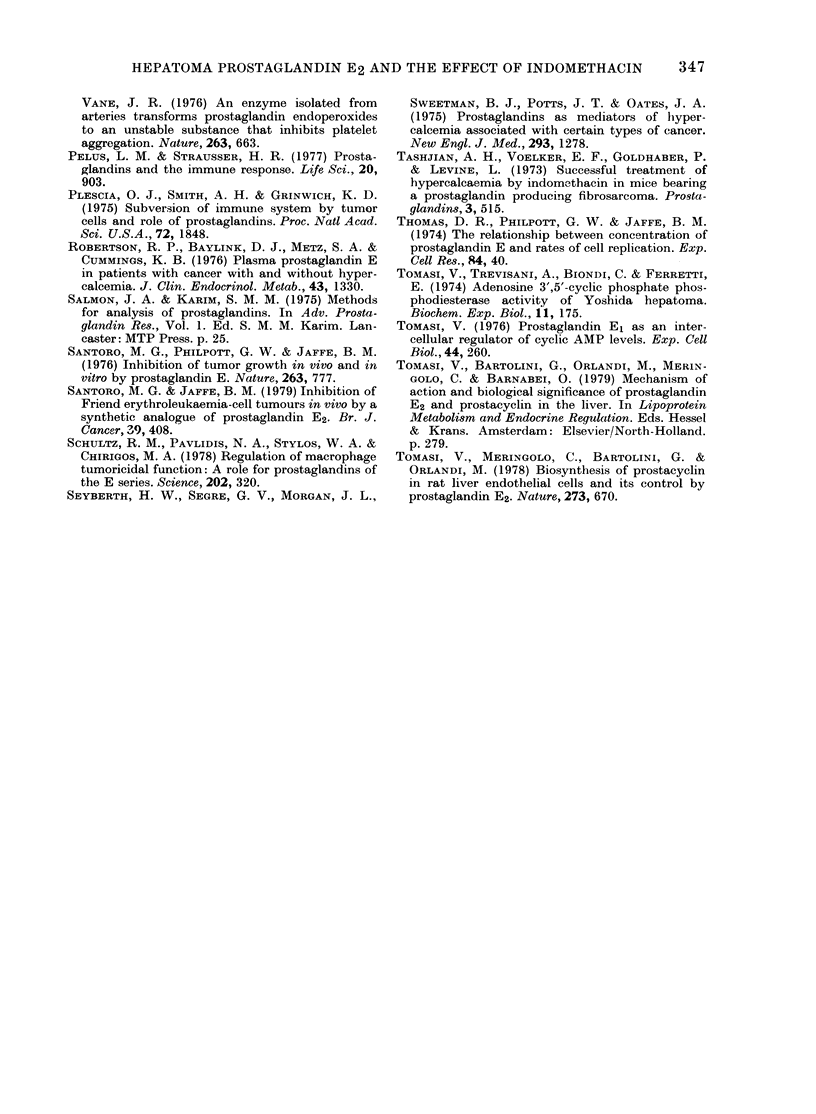


## References

[OCR_00714] Bennett A., Houghton J., Leaper D. J., Stamford I. F. (1979). Cancer growth, response to treatment and survival time in mice: beneficial effect of the prostaglandin synthesis inhibitor flurbiprofen.. Prostaglandins.

[OCR_00721] Brown B. L., Albano J. D., Ekins R. P., Sgherzi A. M. (1971). A simple and sensitive saturation assay method for the measurement of adenosine 3':5'-cyclic monophosphate.. Biochem J.

[OCR_00728] Flower R. J. (1974). Drugs which inhibit prostaglandin biosynthesis.. Pharmacol Rev.

[OCR_00732] Grinwich K. D., Plescia O. J. (1977). Tumor-mediated immunosuppression: prevention by inhibitors of prostaglandin synthesis.. Prostaglandins.

[OCR_00738] Gryglewski R. J., Zmuda A., Korbut R., Krecioch E., Bieron K. (1977). Selective inhibition of thromboxane A2 biosynthesis in blood platelets.. Nature.

[OCR_00744] Hammarström S. (1977). Prostaglandin production by normal and transformed 3T3 fibroblasts in cell culture.. Eur J Biochem.

[OCR_00749] Hong S. L., Wheless C. M., Levine L. (1977). Elevated prostaglandins synthetase activity in methylcholanthrene-transformed mouse BALB/3T3.. Prostaglandins.

[OCR_00755] Kantor H. S., Hampton M. (1978). Indomethacin in submicromolar concentrations inhibits cyclic AMP-dependent protein kinase.. Nature.

[OCR_00764] Levine L., Hong S. L. (1977). Analogues of anthracene, phenanthrene, and benzoflavone inhibit prostaglandin biosynthesis by cells in culture.. Prostaglandins.

[OCR_00770] Lupulescu A. (1978). Enhancement of carcinogenesis by prostaglandins in male albino Swiss mice.. J Natl Cancer Inst.

[OCR_00775] Lynch N. R., Salomon J. C. (1979). Tumor growth inhibition and potentiation of immunotherapy by indomethacin in mice.. J Natl Cancer Inst.

[OCR_00783] Moncada S., Gryglewski R., Bunting S., Vane J. R. (1976). An enzyme isolated from arteries transforms prostaglandin endoperoxides to an unstable substance that inhibits platelet aggregation.. Nature.

[OCR_00788] Pelus L. M., Strausser H. R. (1977). Prostaglandins and the immune response.. Life Sci.

[OCR_00793] Plescia O. J., Smith A. H., Grinwich K. (1975). Subversion of immune system by tumor cells and role of prostaglandins.. Proc Natl Acad Sci U S A.

[OCR_00799] Robertson R. P., Baylink D. J., Metz S. A., Cummings K. B. (1976). Plasma prostaglandin E in patients with cancer with and without hypercalcemia.. J Clin Endocrinol Metab.

[OCR_00816] Santoro M. G., Jaffe B. M. (1979). Inhibition of Friend erythroleukaemia-cell tumours in vivo by a synthetic analogue of prostaglandin E2.. Br J Cancer.

[OCR_00811] Santoro M. G., Philpott G. W., Jaffe B. M. (1976). Inhibition of tumour growth in vivo and in vitro by prostaglandin E.. Nature.

[OCR_00822] Schultz R. M., Pavlidis N. A., Stylos W. A., Chirigos M. A. (1978). Regulation of macrophage tumoricidal function: a role for prostaglandins of the E series.. Science.

[OCR_00828] Seyberth H. W., Segre G. V., Morgan J. L., Sweetman B. J., Potts J. T., Oates J. A. (1975). Prostaglandins as mediators of hypercalcemia associated with certain types of cancer.. N Engl J Med.

[OCR_00835] Tashjian A. H., Voelkel E. F., Goldhaber P., Levine L. (1973). Successful treatment of hypercalcemia by indomethacin in mice bearing a prostaglandin-producing fibrosarcoma.. Prostaglandins.

[OCR_00842] Thomas D. R., Philpott G. W., Jaffe B. M. (1974). The relationship between concentration of prostaglandin E and rates of cell replication.. Exp Cell Res.

[OCR_00868] Tomasi V., Meringolo C., Bartolini G., Orlandi M. (1978). Biosynthesis of prostacyclin in rat liver endothelial cells and its control by prostaglandin E2.. Nature.

[OCR_00854] Tomasi V. (1976). Prostaglandin E1 as an intercellular regulator of cyclic AMP levels.. Exp Cell Biol.

